# Differential gene expression in disease: a comparison between high-throughput studies and the literature

**DOI:** 10.1186/s12920-017-0293-y

**Published:** 2017-10-11

**Authors:** Raul Rodriguez-Esteban, Xiaoyu Jiang

**Affiliations:** 1 Roche Pharmaceutical Research and Early Development, Roche Innovation Center Basel, Grenzacherstrasse 124, 4070 Basel, Switzerland; 2 Biogen, Cambridge, MA USA

## Abstract

**Background:**

Differential gene expression is important to understand the biological differences between healthy and diseased states. Two common sources of differential gene expression data are microarray studies and the biomedical literature.

**Methods:**

With the aid of text mining and gene expression analysis we have examined the comparative properties of these two sources of differential gene expression data.

**Results:**

The literature shows a preference for reporting genes associated to higher fold changes in microarray data, rather than genes that are simply significantly differentially expressed. Thus, the resemblance between the literature and microarray data increases when the fold-change threshold for microarray data is increased. Moreover, the literature has a reporting preference for differentially expressed genes that (1) are overexpressed rather than underexpressed; (2) are overexpressed in multiple diseases; and (3) are popular in the biomedical literature at large. Additionally, the degree to which diseases are similar depends on whether microarray data or the literature is used to compare them. Finally, vaguely-qualified reports of differential expression magnitudes in the literature have only small correlation with microarray fold-change data.

**Conclusions:**

Reporting biases of differential gene expression in the literature can be affecting our appreciation of disease biology and of the degree of similarity that actually exists between different diseases.

**Electronic supplementary material:**

The online version of this article (10.1186/s12920-017-0293-y) contains supplementary material, which is available to authorized users.

## Background

Investigating the differences between diseased and healthy state helps us understand the pathology of diseases and, eventually, treat them. One particular focus of investigation is differentially-expressed genes (DEGs), which involves the identification of genes that are differentially expressed in disease. In pharmaceutical and clinical research, DEGs can be valuable to pinpoint candidate biomarkers, therapeutic targets and gene signatures for diagnostics. While particular gene expression changes may not always translate into consequential biological activity, such data can nonetheless be pooled with other biological data in a high-throughput fashion to create integrated analyses, such as building the target landscape of a disease [1, 2].

Our goal in this study was to compare two widely used sources of DEG information, namely high-throughput microarray expression studies and the scientific literature. For that purpose, we mined the scientific literature and analyzed microarray datasets on a set of diseases to study the similarities and differences of these two types of data within specific biological contexts.

In the scientific literature, information about DEGs is largely found in unstructured form and scattered across publications. It can appear in the form of gradable statements, which are statements that describe a measurement with respect to a baseline, scale or norm [3]. For example, the sentence “The expression of protectin was found to be decreased in the epithelium of patients with ulcerative colitis.” [4] compares the pathological expression of protectin to an implicit baseline, presumably the expression level of protectin in healthy state. Such a sentence describes a “negative regulation of gene expression” as defined by the Gene Regulation Ontology [5].

DEG information can also be found in non-gradable statements in which a comparison is implicit. For example, in the sentence “Expression of the COX-2 enzyme has been reported in animal models of inflammatory bowel disease (IBD) as well as in patients affected by ulcerative colitis and Crohn's disease.” [6] it is implied that there is lack of expression in wild-type animals and healthy patient tissue.

Statements about DEGs in the literature often lack detail or specificity, which is a challenge for human interpretation and for their automatic extraction by computers. Thus, they can refer indistinctly to protein or RNA [7], and use baselines that are not defined or vague. Vagueness is a general feature of natural language and is a special problem with gradable statements [8]. For example, in the sentence “Involucrin [...] is markedly increased in inflammatory skin diseases such as psoriasis.” [9], the magnitude implied by “markedly” is difficult to evaluate. Furthermore, the baseline of the statement is implicit, although it is probably the expression level in healthy skin tissue. Finally, the source of supporting evidence and experimental details, such as the technique employed, is missing in the sentence and in the article in which the sentence appears. Such a statement shows a low level of presented evidence as defined by [10].

In contrast to DEG information found in scientific text, microarray expression data typically appear in structured form in numerical datasets that cover thousands of genes and can be stored in repositories such as the Gene Expression Omnibus (GEO) [11] and ArrayExpress [12]. Such repositories allow the pooling of multiple datasets to create an aggregate view across different experimental settings [13]. While better organized than the literature, microarray expression datasets present their own challenges. The raw expression data from these datasets require processing and quality assessment [14], and resulting expression values convey a relative rather than an absolute measure. Thus, the analysis of microarray expression is usually restricted to identifying expression values with largest change between samples (e.g., [15]) or that change beyond a certain statistically-significant threshold or a fixed fold-change threshold.

An important limitation of microarray expression studies is that they concern only mRNA and not protein, and in particular only whole-cell mRNA [16]. Therefore, they lack the detail and granularity of experimental methods, such as immunohistochemistry, that can describe detailed spatial distributions. Moreover, interpretation of microarray expression results is complicated by the natural variation that exists across biological samples, as well as by differences in technical settings across experiments and laboratories [17]. Finally, microarray expression datasets not stored in standard repositories can be hard to obtain.

The advent of gene expression measurement with RNA sequencing (RNA-seq) technology has affected the number of microarray studies being undertaken. However, in 2016, GEO still released 4945 array expression profiling series (“expression profiling by array”[DataSet Type] AND “gse”[Entry Type]), or about the same quantity released for high-throughput sequencing series (“expression profiling by high throughput sequencing,” *n* = 4894). Moreover, a large trove of microarray studies has been accumulating in GEO over time, with 49,026 array series available (search performed on 2017-2-14). While RNA-seq is increasingly favored for high-throughput expression analysis, modern microarray and RNA-seq platforms produce expression values that are highly correlated and each possesses its own technical advantages [18, 19].

## Methods

For the text mining part of our study, there is no prior work focused specifically on DEGs in disease. The closest work concerns the extraction of population percentages of lymphoma tumors that show expression of a gene in immunohistochemistry experiments [20]. In that work, gene names were tagged using dictionary-matching and a set of rules was devised to identify sentences with potentially relevant information about gene expression. There have also been studies on identifying genes expressed in cell types [21] and anatomical locations [22], or in both [23]. The identification of sentences that describe gene expression, without any other contextual details, has also been addressed as part of more-general event extraction tasks [24, 25].

Our approach was to identify sentences from Medline abstracts that provide information of the type “X is differentially regulated in Y” with respect to healthy controls, with X being a gene and Y a disease. Such information can be mapped to vectors of the type (PMID, X, Y, Δ) where PMID is the corresponding PubMed ID of the abstract and Δ refers to the direction and magnitude of expression change between diseased and healthy states. The values that the vectors (PMID, X, Y, Δ) can take were based, in our case, on the content of the sentences identified, thus coreferences or information from the rest of the document were not considered except in cases of ambiguity in the gene name or anatomical location of the expression. In such cases this information could come from the rest of the abstract if appearing therein. Redundant (PMID, X, Y, Δ) statements were discarded.

Typically, qualifier keywords and phrases (such as “overexpressed,” “decreased expression” and “greatly elevated”) helped determine the value of Δ. The set of possible values for Δ were defined to be the following: {high increase, increase, decrease, high decrease}. We tracked qualifiers that indicated a very large change in expression to assign the Δ values *high decrease* and *high increase*. For example, expression that was described as “greatly elevated” in disease was mapped to *high increase*, while “elevated” or “significantly overexpressed” was mapped to *increase*.

Since DEG information can be conveyed through text in many ways, we devised a generally-inclusive method based on tri-occurrence. We searched first for abstracts mentioning a disease Y and a gene X using disease and human gene annotations from NCBI’s PubTator (download 2016-01-25) [26]. Those abstracts were then split into sentences with the aid of the JULIE Sentence Boundary Detector [27]. For each sentence we detected whether there were mentions of gene X, disease Y (or abbreviation) and trigger word (or substring). The trigger words were selected after [22] to be the following: {express, production, produce, transcription, transcribe}. Finally, the resulting sentences were manually reviewed. Our goal was to produce a sample of sentences that represented an unbiased view of the literature. Undefined expression changes were not considered (e.g. expression described as altered/alteration, aberrant, abnormal, dysregulation, expressed differentially, modulated, discordant). Moreover, names indicating protein complexes or families of proteins or genes that could not be mapped to at most three genes were not considered.

Human microarray expression series relative to each disease were searched in GEO by using the corresponding disease names as keywords. To maintain consistency, all series selected were based on the same platform, Affymetrix Human Genome U133 Plus 2.0 Array (GPL570), and included both diseased tissue samples and normal samples. Unaffected-tissue samples, samples after drug treatment or samples from non-primary disease tissue (such as blood peripheral samples) were not considered. From the series found following these criteria, those with the largest sample size were prioritized.

The series selected for in-depth analysis were: GSE36842 for atopic dermatitis, GSE36807 for Crohn’s disease, GSE13355 for psoriasis and GSE38713 for ulcerative colitis. DEGs were identified using the *limma* Bioconductor R package using Benjamini & Hochberg (false discovery rate) to correct for multiple testing and adjusted *p*-value <0.05. The fold change (FC) in expression was used as a variable filter (cutoff) throughout the study. We did not identify any covariates that required batch correction. Box plots and principal component analysis for each dataset are provided in the supplementary information (see Additional files 1, 2, 3 and 4). For calculating the positive likelihood ratio between microarray data and the literature we took into account only the subset of genes (HUGO gene symbols) shared by both PubTator and GPL570 (*n* = 17,126).

## Results

The focus of our work was on four diseases: Crohn’s disease (CD), ulcerative colitis (UC), psoriasis (PS) and atopic dermatitis (AD). Their choice stemmed partially from their specificity to particular tissues: psoriasis and atopic dermatitis to the skin, Crohn’s disease and ulcerative colitis to the gastrointestinal tract. Another reason for their selection was our interest in exploring similar diseases that are often compared to each other, in our case the pairs PS-AD and UC-CD. We collected DEG statements from the literature and microarray datasets concerning these four diseases (see Methods), focusing only on the main affected tissues (e.g., we discarded serum measurements). We then compared the data reported in the literature with the information contained in microarray datasets.

Through our text mining approach, we created a sample of DEG statements coming from 200 Medline abstracts for AD, 308 for CD, 429 for PS and 273 for UC. These statements concerned 173 unique genes for AD, 240 for CD, 327 for PS and 285 for UC*.* (The text mining results are available as supplementary information, see Additional file 5.) The microarray datasets presented different quantities of DEGs depending on fold change (FC) filtering. For example, for |FC| > 2, 110 unique genes were differentially expressed in AD, 92 in CD, 998 in PS and 2339 in UC.

### Overexpression is more reported than underexpression

**Fig. 1:**
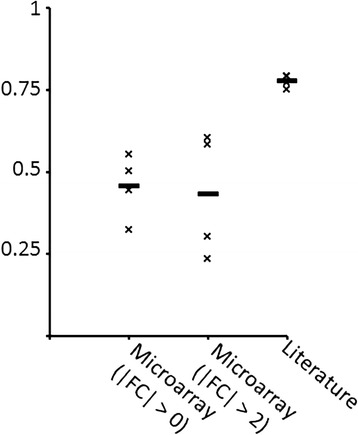
Ratio of overexpressed vs. underexpressed unique DEGs in microarray datasets vs. the literature. |FC| > *n* indicates microarray DEGs with absolute fold change above *n*

As can be seen in Fig. 1 and Table 1, DEG reports favor overexpressed genes 3-4 times more than underexpressed genes. Intriguingly, the magnitude of this bias does not differ much between diseases. Microarray expression data shows no such systematic imbalance.

**Table 1:** Percentage of overexpressed vs. underexpressed unique DEGs in microarray data and the literature. |FC| > *n* indicates microarray DEGs with absolute fold change above *n*

	Microarray		Literature		Microarray		Literature
	|FC| > 0	|FC| > 2			|FC| > 0	|FC| > 2	
AD				PS			
Overexpressed	44.6%	23.6%	79.4%	Overexpressed	55.5%	58.5%	79.2%
Underexpressed	55.4%	76.4%	20.6%	Underexpressed	44.5%	41.5%	20.8%
CD				UC			
Overexpressed	32.6%	30.4%	75.2%	Overexpressed	50.4%	60.6%	77.4%
Underexpressed	67.4%	69.6%	24.8%	Underexpressed	49.6%	39.4%	22.6%

To simplify the discussion, the focus in the next sections is on overexpressed genes, for which there exist more data in the literature.

### The reporting of high overexpression correlates with the reporting of overexpression and only weakly with microarray fold change

**Fig. 2:**
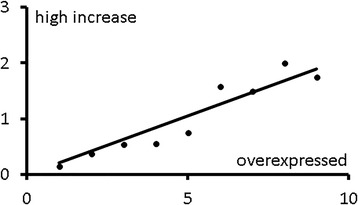
Relation between overexpression mentions in the literature and the subset of those which are *high increase*. The figure shows the relation between gene overexpression mentions and mean number of *high increase* mentions for genes with up to nine overexpression mentions. Slope of the zero-y-intercept trend line is 0.21 and its associated *r* ^*2*^ is 0.89

The more a gene is mentioned as overexpressed in a disease the more likely it will be mentioned as highly overexpressed (highly increased) in the same disease (Fig. 2). One potential explanation for this is that highly overexpressed genes are the focus of more scrutiny due to their presumed heightened biological relevance. There are examples of this phenomenon that can be observed in the literature. Such is the case of gene S100A7 in psoriasis, which first raised interest as a highly expressed gene in psoriatic skin [28]. On the other hand, it is also possible that overexpressed genes that are often studied end up being considered highly overexpressed as the result of sheer multiple testing. Using the data available in our study we used a simple linear model to disentangle this question:

1 $$ high\kern0.28em increase\kern0.28em mentions\sim f\kern0.28em \left( FC; increase\kern0.28em mentions\right)=\alpha \cdot FC+\beta \cdot increase\kern0.28em mentions+\gamma . $$

Through this model we saw that, once we account for the fact that a gene has been mentioned as overexpressed in the literature, the microarray FC value still influences whether it will be described as highly increased or not. The α coefficient for the linear model varies from smallest for UC to largest for PS and is in all cases smaller than 0.01. Thus, vague *high increase* statements are only to a small degree linked to the FC value from microarray data.

### To be reported as overexpressed a gene’s popularity is more important than its fold change

**Fig. 3:**
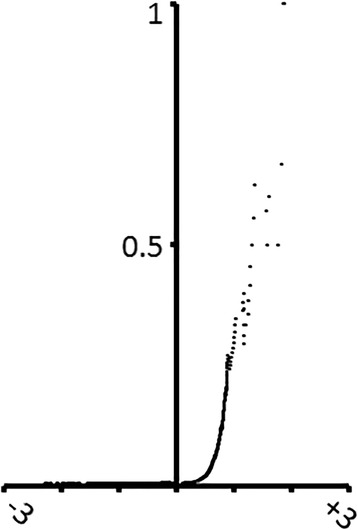
Cumulative probability of a gene being reported as overexpressed in AD given its microarray FC. The abscissa corresponds to microarray FC and the ordinate to the cumulative probability of a gene being reported as overexpressed when its associated microarray FC is above a certain value, *p(overexpression in AD | FC in AD > x)*

One way to see the relation between microarray FC and the literature is by looking at the probability that a gene will be reported as overexpressed for FC values above a certain threshold. As can be seen in Fig. 3 for the case of AD, the cumulative probability increases with FC, which means that genes associated to higher FCs are more likely to be reported as overexpressed in the literature.

However, there is also a correlation between the frequency with which a gene is mentioned as overexpressed and its popularity in the overall biomedical literature, as can be seen in Table 2. Thus, genes that are reported as overexpressed in a disease tend to be popular in the biomedical literature at large. Microarray data FC, on the other hand, exhibit lower correlation with overexpression reporting or none. Only the PS and UC microarray datasets showed statistically significant correlation and of smaller magnitude than that associated to popularity.

**Table 2:** Pearson correlation coefficient (r) between a gene’s popularity (total number of mentions in the biomedical literature) and its overexpression in a disease according to the literature (0 = not mentioned, 1 = mentioned) or to microarray data (0 = not overexpressed, 1 = overexpressed)

		AD	CD	PS	UC
Literature		0.167*	0.208*	0.219*	0.222*
Microarray	FC > 0	0.003	0.002	0.036*	0.031*
	FC > 2	0.002	−0.002	0.026*	0.060*

Asterisks indicate statistically significant values

To further test the relation between overexpression reports, microarray FC and popularity; we created a linear model in which overexpression mentions were a function of the variables log_2_FC and popularity:

2 $$ increase\kern0.28em mentions\sim f\left({log}_2 FC; popularity\right)=\alpha \cdot {log}_2 FC+\beta \cdot popularity+\gamma . $$

Both of these variables turned out to be significant for each disease, except in the case of PS, for which log_2_FC was not significant. Thus, a gene’s chances to be mentioned as overexpressed can increase both with its microarray FC value and with its popularity in the general literature, but popularity has greater influence.

### In terms of overexpression, the literature shows diseases to be more similar than microarrays do

**Fig. 4:**
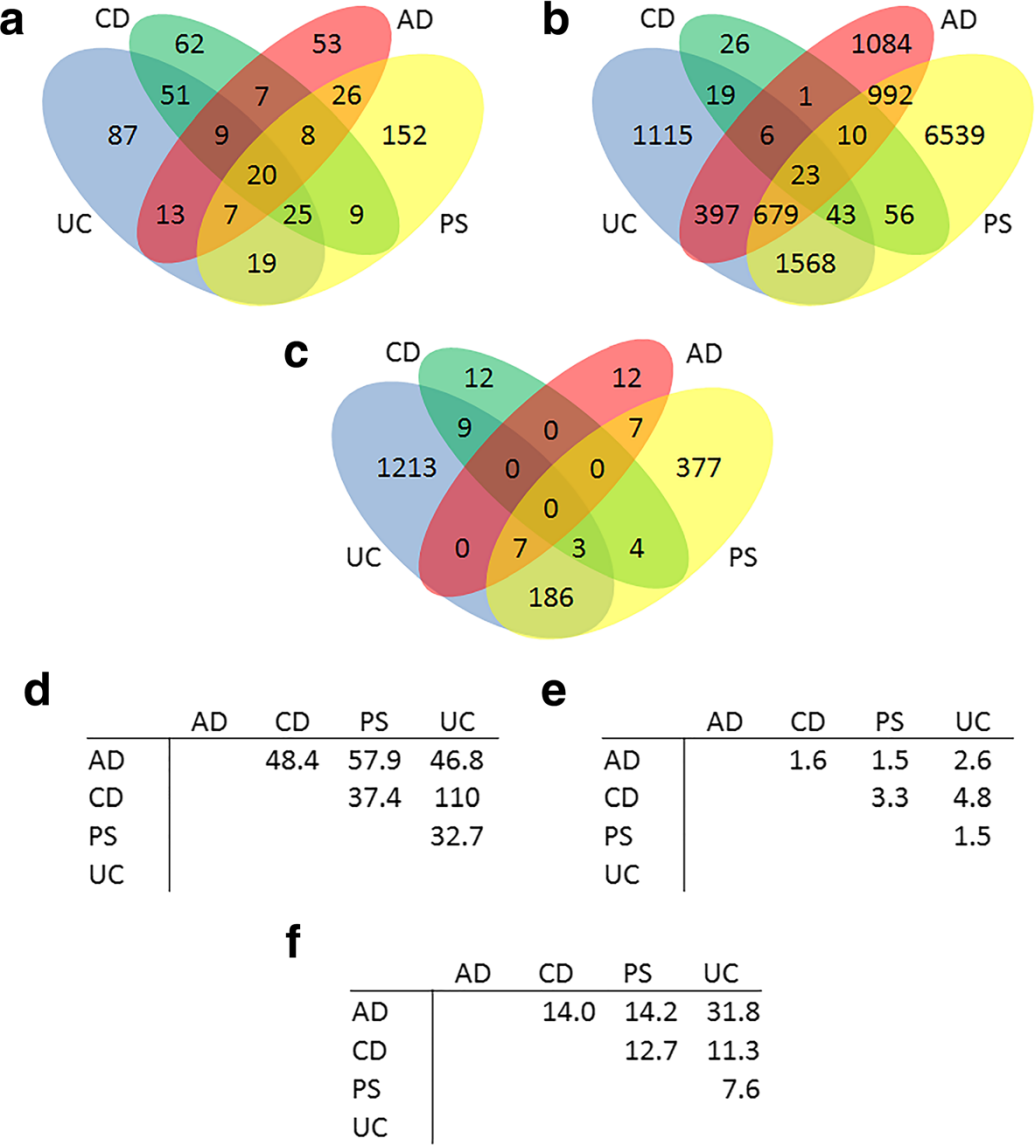
Number of overexpressed genes for each disease. Number of overexpressed genes for each disease (**a**) as reported in the literature and (**b** and **c**) as appearing in microarray datasets (FC > 0 and FC > 2, respectively). The tables show the LR+ for genes overexpressed in one disease (table headers) that are overexpressed in another disease (row names) based on (**d**) the literature or (**e**) microarray data with FC > 0 or (**f**) FC > 2

As can be seen in Fig. 4, from the point of view of gene overexpression, similarities between any pair of diseases are generally higher in the literature than in microarrays. This can be quantified using the positive likelihood ratio (LR+) following the equation:

3 $$ LR+\left({Y}_1/{Y}_2\right)=\frac{p\kern0.28em \left( overexpression\kern0.28em in\kern0.28em {Y}_1\kern0.28em |\kern0.28em overexpression\kern0.28em in\kern0.28em {Y}_2\operatorname{}\right)}{p\kern0.28em \left( overexpression\kern0.28em in\kern0.28em {Y}_1|\kern0.28em no\kern0.28em overexpression\kern0.28em in\kern0.28em {Y}_2\operatorname{}\right)} $$

For example, based on microarray data with FC > 0 cutoff, the LR+ for AD based on CD (AD/CD) is 1.6, which means that a gene is 1.6 times more likely to be overexpressed in AD when that gene is overexpressed in CD. Meanwhile, in the literature, the value of LR+(AD/CD) is 48.4, which is much higher. Thus, the literature is enriched for genes that are overexpressed in more than one disease, as can be seen in Fig. 4. Overall, for FC > 0, microarray datasets show LR+ values between 1 and 5 while the literature yields LR+ values between 32 and 110. For FC > 2, on the other hand, microarray data shows higher LR+ values, although still lower than those for the literature.

The differences in LR+ between the literature and microarray data are larger when it comes to genes reported to be overexpressed in three out of our four diseases. The LR+ for microarray data with FC > 0 ranges between 1 and 5 (mean ~ 2.6) while it ranges between 40 and 91 (mean ~ 64) for the literature. Finally, for genes overexpressed in all four diseases the LR+ for microarray data with FC > 0 ranges between 1 and 7 (mean ~ 3.4) while for the literature it ranges between 75 and 122 (mean ~ 110).

Naturally, certain disease pairs will share more overexpressed genes due to biological similarities. However, we found that the level of similarity between diseases differs depending on whether microarray data or the literature was considered. For example, taking microarray data with FC > 0 cutoff as a “true” baseline, the literature would be overstating the similarity of PS and AD the most, while the similarities between PS and CD would be the least emphasized. Thus, it is possible that the similarities between PS and CD have received insufficient attention (see for example [29]) in comparison to the similarities between PS and AD, if microarray data is to be used as guidance.

### As the microarray fold-change cutoff increases, microarray data and the literature increase in resemblance

The LR+ can also help us determine further the relationship between overexpression in the literature and in microarrays. We can compute the LR+ of a gene being overexpressed in the literature when it is overexpressed in microarray data and vice versa. Our interest is in knowing whether the odds of a gene being overexpressed in one of the sources change when it is known to be overexpressed in the other source.

**Fig. 5:**
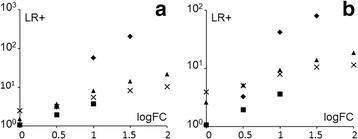
Positive likelihood ratio given microarray data and the literature. Positive likelihood ratio (LR+) of (**a**) microarray data given the literature and (**b**) the literature given microarray data for different values of log_2_FC threshold and for each disease: AD (diamonds), CD (squares), PS (triangles), UC (crosses). The higher the LR+ the more likely one data source can predict another one

Our finding was that the LR+ depends on the FC cutoff chosen. For example, the LR+ of microarray overexpression for FC > 0 given the literature (and vice versa) is not significant for AD and CD. For PS and UC the LR+ is significant and ranges between 1.5 and 4 (see Fig. 5). Thus, the information conveyed by these two sources can be quite distinct when choosing a FC > 0 cutoff. In the case showing highest LR+ (UC), the probability of a gene being overexpressed in the microarray dataset goes up from 21 to 50% when the literature states that it is overexpressed. The probability of a gene being overexpressed in the UC literature goes up from 0.09 to 0.34% when it is overexpressed in the microarray dataset.

A different picture arises with increased FC thresholds, as can be seen in Fig. 5. The LR+ then increases substantially, which means that the literature becomes more related to microarray data as the FC threshold increases. This is probably due to the fact that, as has been already stated, the probability that a gene is mentioned as overexpressed in the literature increases with higher microarray FC.

### Differences between microarray data and the literature translate into alternative views of the underlying disease biology

**Fig. 6:**
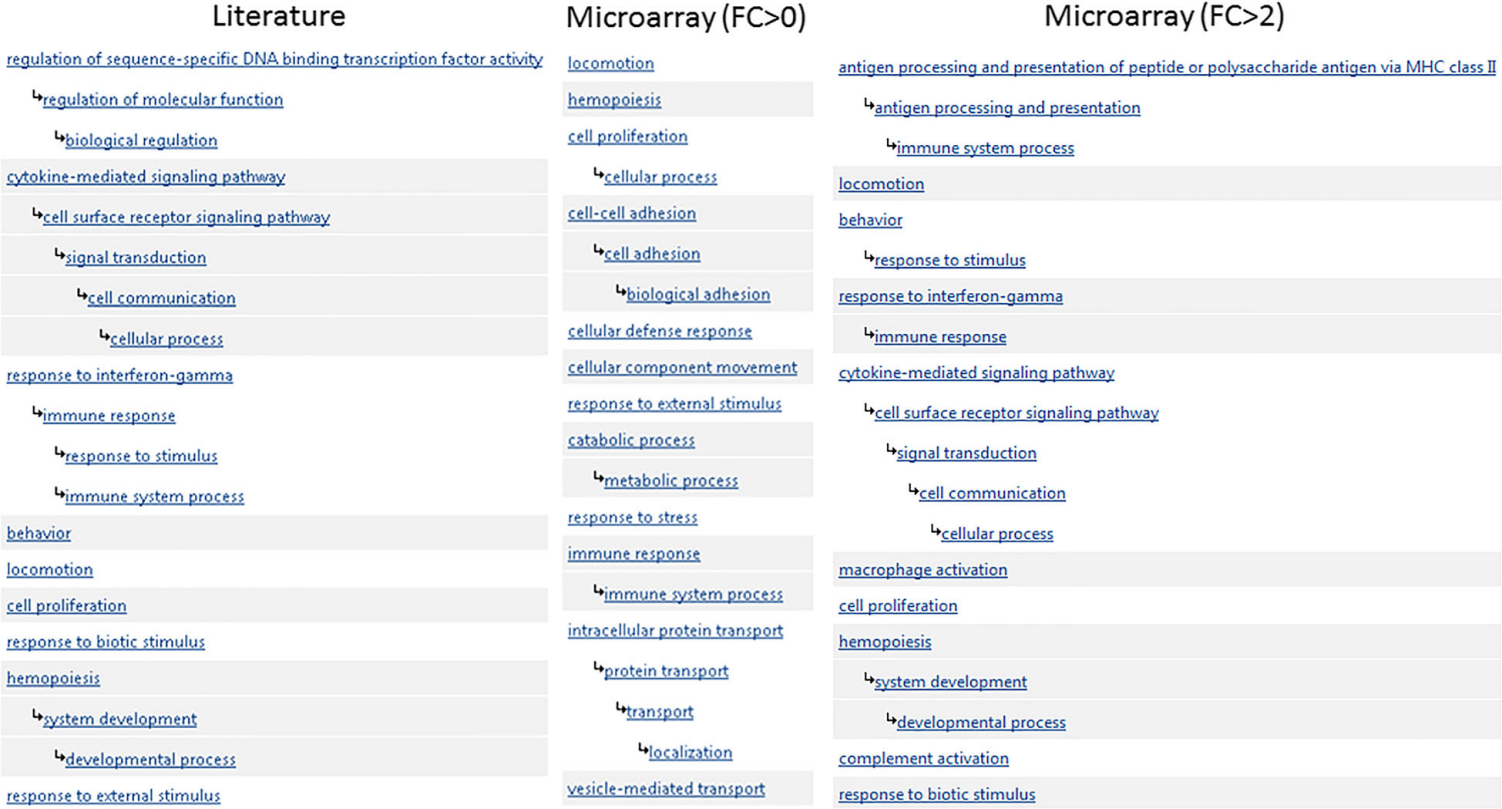
Statistically overrepresented Gene Ontology functional classes. Top-20 statistically overrepresented Gene Ontology functional classes based on overexpressed genes in the UC literature (left) and in the UC microarray dataset (right)

As could be expected, the differences that have been described between microarray and literature data translate into different representations of the pathological processes that characterize each disease. To measure this quantitatively, we looked at the level of enrichment of Gene Ontology (GO) functional classes associated to the genes overexpressed in microarray data and in the literature. Figure 6 shows the top 20 statistically overrepresented GO functional classes in microarray and literature data for UC based on the PANTHER statistical overrepresentation test with Bonferroni correction [30]. For UC and FC > 0, 16 functional classes were shared between the 38 overrepresented in the literature and the 36 overrepresented in the microarray dataset. For PS and FC > 0, on the other hand, only the “unclassified” functional class was shared between the 17 overrepresented in the microarray dataset and the 15 overrepresented in the literature.

For FC > 2, the similarities between microarray data and the literature were greater. For UC there were 28 shared functional classes between the 47 overrepresented in the microarray dataset and the 38 overrepresented in the literature. For PS, there were 11 shared functional classes between the 28 overrepresented in the literature and the 18 overrepresented in the microarray dataset.

## Discussion

Our goal was to explore the relationship between microarray expression data and the expression data reported in the literature because in our daily work both of these data sources are used as complementary sources of information. From the therapeutic point of view, for example, every DEG in disease is a potential point of intervention or target. Thus, the sole use of microarray data or of the literature could lead to missing out on potential targets that appear in one source and not the other. For instance, EGFR does not appear upregulated in the PS microarray dataset, while it is one of the most frequently mentioned upregulated genes in the PS literature dataset. On the other hand, defensin beta 4B (DEFB4B) does not appear in the PS literature dataset despite showing the second-highest level of overexpression in the PS microarray dataset.

Our strategy for gathering microarray data was to select one dataset for each disease of interest, each dataset created with the same platform to avoid variability across manufacturers. For literature data, our approach was to gather a representative sample of the literature, rather than to create an exhaustive representation. We, moreover, focused on abstracts, rather than on full text articles, due to limited full text availability. Thus, the true number of statements regarding differential expression in the literature is larger than what is reported here.

The fact that more literature results were oriented towards overexpression than underexpression, unlike in microarray data, indicates a scientific bias towards reporting overexpression. This bias could be related to the fact that most drugs are inhibitors and therefore an overexpressed gene is more likely to represent a potential target. Since, in principle, downregulation may have as much functional importance in disease as upregulation, this bias could be distorting in our understanding of diseases.

We also noted that popular genes tend to be more often described in the literature as overexpressed in disease, an effect that is much milder or non-existent for overexpressed genes from microarray data. This could explain partially why differential expression similarities between diseases are higher within the literature in comparison to microarray data. The quest for higher research impact could be one of the drivers for the additional attention paid to popular genes [31–33], leading to further amplification of their presumed biological importance beyond actual biological evidence.

Our analysis also hints that our perception of the level of similarity between certain diseases could be biased by general properties of the diseases that are not reflected in the expression data. Thus, PS and AD, which share anatomical location, appear more similar in the literature than UC and AD, contrary to what is reflected in microarray data.

We also found that microarray data and the literature can produce divergent views of the pathological mechanisms driving diseases depending on the fold-change cutoff. For FC > 0, the functional classes associated to overexpressed genes in the literature can be very different from those associated to microarray data. As the threshold for FC increases, the similarity between the literature and microarray data increases, which is then reflected in higher LR+ values and overlapping functional classes.

One explanation for the divergences between microarray data and the literature comes obviously from the differences in experimental settings. Expression data from the literature stem from a variety of sources involving methods such as immunohistochemistry, flow cytometry, in situ hybridization, RT-PCR, next-generation sequencing--and also microarrays. Each of these sources differs in level of granularity and molecule measured (e.g. mRNA vs. protein). On the other hand, even though all microarray data in our study came from the same platform from the same manufacturer, and each dataset was created within a single research study, microarray data variability has been shown to be a challenge for reproducibility [34–37].

Moreover, because experiments in the literature can be more fine-grained than microarray studies, it is possible that a gene might be found to be upregulated in some parts of a diseased tissue and downregulated in others, confounding the simplified representation used here and hampering comparisons with microarray data.

One additional aspect not considered in this study was the historical dimension. High-throughput techniques have been gaining in popularity only recently; therefore older publications would have been less affected by findings coming from high-throughput studies.

## Conclusion

At the start of this study we had the expectation that there would be certain biases in the literature in comparison to microarray data. The literature evidently has a focus that is, at the very least, biased by past research history, which does not affect microarray data. Our goal was to quantify this bias, using microarray data as the unbiased “ground truth.” However, we did not expect that the relationship between microarray data and the literature could be dependent on FC cutoff (which in retrospect appears to be naïve), and therefore that we should not necessarily consider microarray data a ground truth that the literature only partially represents.

The use of an FC threshold does not in principle have a fixed biological meaning and its link to biological activity can change from gene to gene. Moreover, different FC thresholds yield different outcomes from an expression study [38]. Based on our work, the literature has a closer connection with microarray expression data filtered with higher FC thresholds, which means that it may not track biological phenomena appropriately when the FC thresholds do not actually separate meaningful and non-meaningful expression changes.

## Additional files

Additional file 1: Boxplot and PCA for AD. Boxplot and principal component analysis for the GSE36842 study. (TIFF 189 kb)

Additional file 2: Boxplot and PCA for CD. Boxplot and principal component analysis for the GSE36807 study. (TIFF 120 kb)

Additional file 3: Boxplot and PCA for PS. Boxplot and principal component analysis for the GSE13355 study. (TIFF 100 kb)

Additional file 4: Boxplot and PCA for UC. Boxplot and principal component analysis for the GSE38713 study. (TIFF 226 kb)

Additional file 5: Text mining results. Curated results produced by the text mining algorithm. (XLSX 162 kb)

## Abbreviations

AD: Atopic dermatitis
DEFB4B: Defensin beta 4B
DEG: Differentially-expressed gene
FC: Fold change
GEO: Gene Expression Omnibus
GO: Gene Ontology
IBD: Inflammatory bowel disease
LR: Likelihood ratio
NCBI: National Center for Biotechnology Information
PCA: Principal component analysis
PMID: PubMed ID
PS: Psoriasis
RA: Rheumatoid arthritis
RNA-seq: RNA sequencing
UC: Ulcerative colitis
