# Supplementary figures and images for: Differential gene expression in disease: a comparison between high-throughput studies and the literature
Source: BMC Med Genomics. 2017 Oct 11;10:59. doi: 10.1186/s12920-017-0293-y (PMC5637346; doi:10.1186/s12920-017-0293-y)

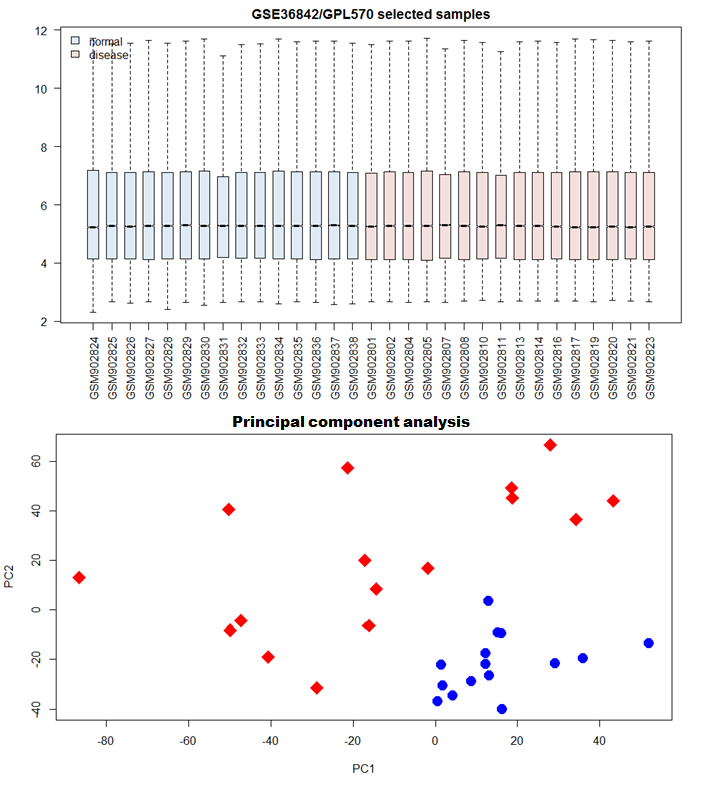

Supplement: Supplementary file 1 — Boxplot and PCA for AD. Boxplot and principal component analysis for the GSE36842 study. (TIFF 189 kb) [file 12920_2017_293_MOESM1_ESM.tif]

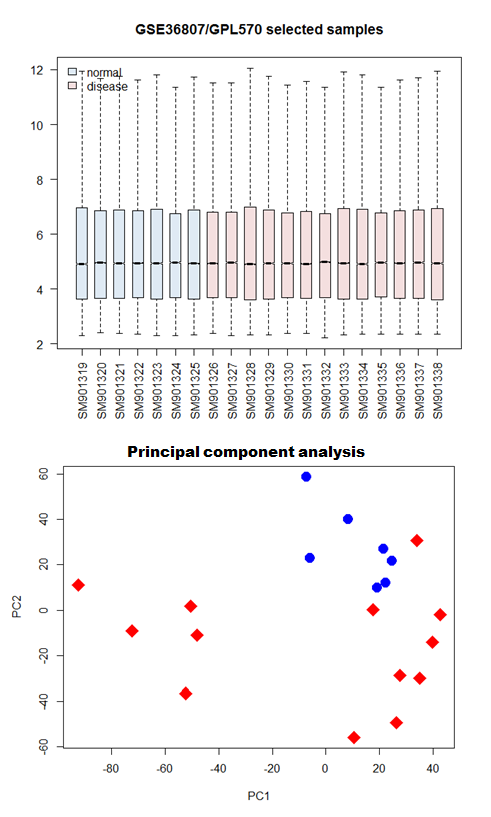

Supplement: Supplementary file 2 — Boxplot and PCA for CD. Boxplot and principal component analysis for the GSE36807 study. (TIFF 120 kb) [file 12920_2017_293_MOESM2_ESM.tif]

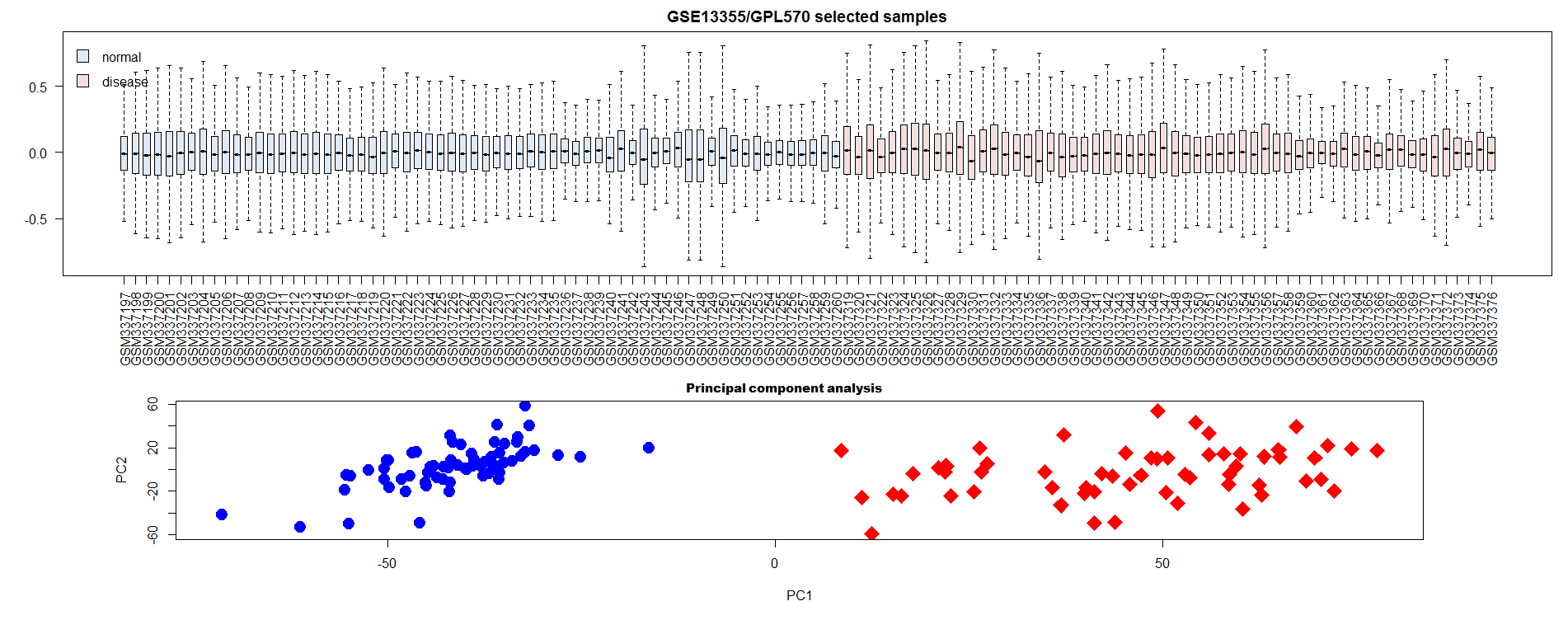

Supplement: Supplementary file 3 — Boxplot and PCA for PS. Boxplot and principal component analysis for the GSE13355 study. (TIFF 100 kb) [file 12920_2017_293_MOESM3_ESM.tif]

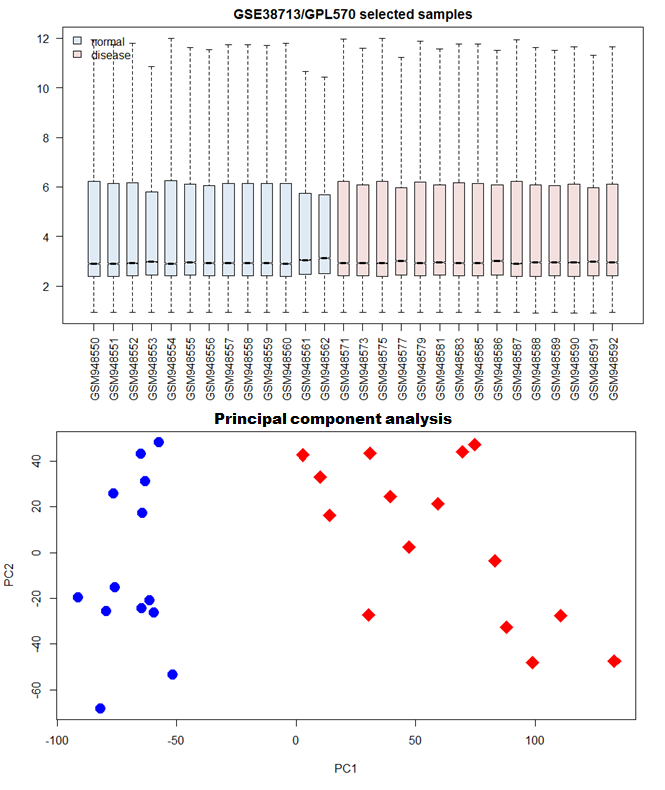

Supplement: Supplementary file 4 — Boxplot and PCA for UC. Boxplot and principal component analysis for the GSE38713 study. (TIFF 226 kb) [file 12920_2017_293_MOESM4_ESM.tif]
